# Integrated assessment of predicted MHC binding and cross-conservation with self reveals patterns of viral camouflage

**DOI:** 10.1186/1471-2105-15-S4-S1

**Published:** 2014-03-19

**Authors:** Lu He, Anne S De Groot, Andres H Gutierrez, William D Martin, Lenny Moise, Chris Bailey-Kellogg

**Affiliations:** 1 Department of Computer Science, Dartmouth College, Hanover, NH, USA; 2 Institute for Immunology and Informatics, University of Rhode Island, Providence, RI, USA; 3 EpiVax Inc., Providence, RI, USA

## Abstract

**Background:**

Immune recognition of foreign proteins by T cells hinges on the formation of a ternary complex sandwiching a constituent peptide of the protein between a major histocompatibility complex (MHC) molecule and a T cell receptor (TCR). Viruses have evolved means of "camouflaging" themselves, avoiding immune recognition by reducing the MHC and/or TCR binding of their constituent peptides. Computer-driven T cell epitope mapping tools have been used to evaluate the degree to which particular viruses have used this means of avoiding immune response, but most such analyses focus on MHC-facing 'agretopes'. Here we set out a new means of evaluating the TCR faces of viral peptides in addition to their agretopes, integrating evaluations of both sides of the ternary complex in a single analysis.

**Methods:**

This paper develops what we call the *Janus Immunogenicity Score (JIS)*, bringing together a well-established method for predicting MHC binding, with a novel assessment of the potential for TCR binding based on similarity with self. Intuitively, both good MHC binding and poor self-similarity are required for high immunogenicity (i.e., a robust T effector response).

**Results:**

Focusing on the class II antigen-processing pathway, we show that the JIS of T effector epitopes and null or regulatory epitopes deposited in a large database of epitopes (Immune Epitope Database) are significantly different. We then show that different types of viruses display significantly different patterns of scores over their constituent peptides, with viruses causing chronic infection (Epstein-Barr and cytomegalovirus) strongly shifted to lower scores relative to those causing acute infection (Ebola and Marburg). Similarly we find distinct patterns among influenza proteins in H1N1 (a strain against which human populations rapidly developed immunity) and H5N1 and H7N9 (highly pathogenic avian flu strains, with significantly greater case mortality rates).

**Conclusion:**

The Janus Immunogenicity Score, which integrates MHC binding and TCR cross-reactivity, provides a new tool for studying immunogenicity of pathogens and may improve the selection and optimization of antigenic elements for vaccine design.

## Background

**Figure 1:**
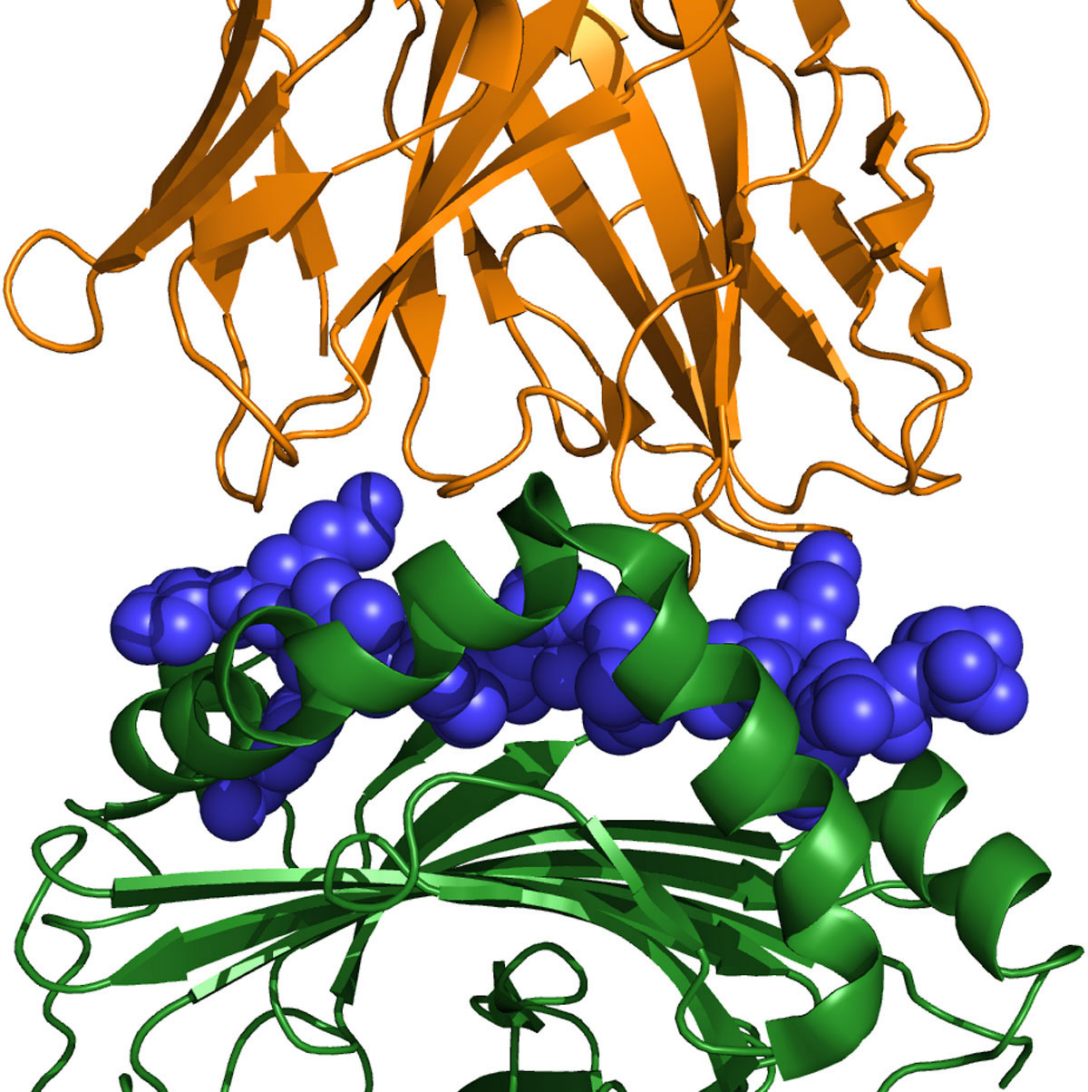
**The two-faced T cell epitope**. The ternary MHC: peptide epitope: TCR complex drives the class II immune response (structure from pdb id 1fyt; rendered with PyMol (Schrödinger, LLC)). Some of the epitope side-chains (blue, spacefill) interact with the MHC (green, cartoon), while others interact with the TCR (orange, cartoon). A TCR may recognize many peptides that have similar TCR-facing sequences but different MHC-facing sequences, as long as the peptide still binds the same MHC. We develop an integrated scoring mechanism that accounts for both aspects of immune recognition, combining the EpiMatrix epitope predictor (to identify peptides that are good MHC binders) with an assessment of cross-reactivity with human genome (to identify peptides that are unlike self and thus more likely to be recognized by TCR from effector T cells).

Cellular immune defenses against infectious diseases are dependent on the immune system's ability to identify and control invading pathogens without causing too much collateral damage. To accomplish that, the immune system employs complex networks of interacting cells and molecules. In one key network, Antigen Presenting Cells (APCs) process and then present to circulating T cells peptides that they have derived from pathogenic proteins. An APC loads such a peptide, called a T cell epitope, onto its Class II Human Leukocyte Antigen (HLA), also known as Major Histocompatibility Complex (MHC). Engagement of the T Cell Receptor (TCR) with the T cell epitope:MHC II complex on the surface of the APC triggers a cascade of events leading to activation of the T cell response. Thus the ternary MHC:T-cell epitope:TCR complex connects the APC to the responder T cell [1,2] (Figure 1).

This antigen-presenting pathway is constantly processing proteins that have been sampled from the extracellular environment. In the case of viruses and bacteria, peptides (derived from their constituent proteins) that bind MHC and TCR are recognized as foreign and trigger an immune response. T helper (MHC class II-restricted) epitopes within viral and bacterial proteins are a necessary component of immune protection against these pathogens since the presentation of a T-cell epitope in the context of MHC Class II molecules is essential for a B-cell to be stimulated and produce high-affinity and high-titer antibodies. Since this interaction is the one that initiates a specific immune response against the pathogen, one means by which pathogens evade immune response is to present proteins with lower immunogenic potential [3]. (Similar pressure results from the class I pathway wherein CD8+ cytotoxic T lymphocytes recognize and destroy infected cells, but this paper focuses on class II.)

The recognition of a peptide depends on both the MHC face and the TCR face (Figure 1), giving a pathogen two different routes for escape by modification of its immunogenic peptides. In order to assess immunogenic potential and study escape on a genomic scale, it is necessary to apply computational tools. On the MHC-binding side, a number of algorithms have been developed and used to map T cell epitopes (both MHC Class I and Class II-restricted) within protein molecules of various origins (see review and a current list of immunoinformatics tools in reference [4]). Many algorithms rely on the linear nature of T cell epitopes as it simplifies modeling their interactions with MHC in defined binding pockets, which contrasts with the complexities of B cell epitope prediction that involves the interaction of discontinuous antigen sequences with highly variable complementarity determining regions of antibodies. Such *in silico *predictions of T-helper epitopes have been successfully applied to the design of vaccines [5,6] and to the selection of epitopes in studies of autoimmunity [7]. We use here the EpiMatrix system, a suite of epitope mapping tools that has been validated over the course of more than a decade, both *in vitro *and *in vivo *(for example, see references [8-14]).

Computational T-cell epitope mapping tools have been used to evaluate the degree to which selected pathogens have reduced their epitope content in order to evade immune response. For example, one study of CD8+ T cell epitopes in viruses showed that those infecting humans contained fewer CD8+ T-cell epitopes than non-human-host viruses, with human-host viral proteins expressed earlier in the life cycle having a lower epitope density than those expressed later [15]. Similarly, a study of CD8+ T-cell epitopes in bacteria demonstrated that host cytosol-exposed proteins showed clear escape mutations and low epitope density while proteins not translocated to the cytosol showed no escape mutations and some of them were found to have high epitope density [16]. We have also previously performed large-scale analyses of human proteins to determine whether the number of T-cell epitopes in secreted or extracellular proteins is reduced in comparison with other non-secreted proteins [17], and found evidence supporting our hypothesis that highly prevalent secreted proteins that are present in the serum are 'naturally deimmunized' with respect to other, less prevalent and more internal proteins.

Characterizing and making predictions for the TCR side of recognition is substantially harder than for the MHC side, due to the essentially unpredictable variability in the complementarity determining regions of the TCR. However, in the context of pathogenic immune escape, there is one critical aspect that is more tractable for analysis: similarity to self. Autologous proteins are also processed and presented by antigen presenting cells, but they do not usually trigger immune responses by T cells due to mechanisms of central and peripheral tolerance. For T cells, initial self/non-self discrimination occurs in the thymus during neonatal development when medullary epithelial cells express tissue-specific self-proteins and present the epitopes therein to immature T cells. T cells recognizing self-antigens with high affinity are deleted; auto-reactive T cells with moderate affinity may escape deletion and be converted to function as 'natural' regulatory T cells (Treg) cells [18]. While central deletion of auto-reactive T cells is the primary means by which tolerance is established, some auto-reactive T cells escape this mechanism, potentially contributing to autoimmunity. Natural regulatory T cells that recognize epitopes from autologous proteins circulate in the periphery, suppressing anti-self immune response. Both thymic-derived (natural or nTreg) and induced (iTreg) regulatory T cells bearing T cell receptors that recognize self-antigens are involved in regulating autoimmunity [19].

Assuming then that autologous proteins are less likely to generate T effector responses, an immune escape mechanism for a pathogen is to ensure that MHC-binding peptides within its proteins present human-like TCR faces. This is a more relaxed requirement than full "humanization" (ensuring that the pathogenic and human peptide are identical); instead, the peptides only need to be *cross-reactive *(binding the same TCRs). Cross-reactivity is an intrinsic characteristic of the T cell receptor, in that each TCR can potentially interact with many different T cell epitopes [20-22]. T cell epitope cross-reactivity is also critical to many aspects of T cell biology, including positive and negative T cell selection in the neonatal thymus [23,24].

Thus one means of immune escape by viruses may be to present T cell epitopes recognized by nTregs or iTregs. TCR-level cross-reactivity with autologous proteins is of great interest in general as T cells responding to a foreign protein may have diminished or altered type of immune response, and auto-reactive T cells may be triggered by this cross-reactivity [25]. We are particularly interested in understanding to what extent cross-reactivity might differ between commensal viruses (e.g., Epstein-Barr and cytomegalovirus) that can continuously infect humans for years while demonstrating very limited pathology ("hit-and-stay" viruses, as described by Hilleman [3]), as opposed to other ("hit-and-run") viruses that are known to cause significant immunopathology and death (e.g., Ebola and Marburg). We also wish to determine whether T cell epitopes from any viral source might be less likely to trigger an immune response when tested in humans, if cross-reactivity with self was found to be present.

Computational analyses of sequence similarity between predicted epitopes in pathogens and in the human genome have been used to study TCR-level cross-reactivity. Previous studies [26] showed for class I epitopes that the recognition of a peptide-MHC by the T-cell receptor is flexible, and as a result, about one-third of non-self peptides are expected to be indistinguishable (by T-cells) from autologous peptides. T-cells are expected to remain tolerant to self, leading to the creation of "holes" in the immune recognition repertoire for cross-reactive foreign epitopes. The overlap with self increases the need for efficient self-tolerance, as many self-similar non-self peptides could initiate an autoimmune response.

So as to enable broad computational studies of cross-reactivity, we recently developed a new immunoinformatic tool, JanusMatrix [27]. This tool, enabled by the EpiMatrix T cell epitope-mapping platform described above, is designed to easily and efficiently identify potential cross-reactivity among T cell epitopes computationally mapped for human pathogens, the human genome, and the human microbiome. An initial evaluation of validated Treg epitopes showed that their TCR-facing residues had statistically greater TCR cross-reactivity with human sequences. Teff epitope and Treg epitope cross-reactivities with human sequences were also different from random nine-mers. We postulated that some of the overlap between human pathogens and the human genome might be due to co-opting of human-genome cross-reactivity by human pathogens to escape immune response.

Building on this observation, this paper presents a new framework for quantitatively characterizing immunogenicity risk, unifying the MHC and TCR sides of immune recognition. The Janus Immunogenicity Score (JIS) provides a probabilistic assessment of the potential of a peptide to induce a T effector response, combining evaluation of MHC binding potential with identification of cross-reactivity against the human genome. We show that JIS is predictive of Teff data deposited in the immune epitope database (IEDB). We then show that it reveals patterns of camouflage that differ between hit-and-stay viruses that cause chronic infection (Epstein-Barr and cytomegalovirus) and hit-and-run ones that cause acute infection (Ebola, Marbug, and plant-host viruses). We also consider a wide range of other human-host viruses and see that most appear quite similar in terms of their relative camouflage, while some are striking outliers. We finally zoom in and apply a similar analysis to specific influenza proteins from different strains, characterizing their relative visibility to the human immune system.

## Methods

### Janus immunogenicity score

The Janus immunogenicity score *J*(*p*; *A*) is defined for a peptide *p *with respect to a set *A *of MHC alleles. Throughout this manuscript, we use for *A *eight common representative alleles (DRB1*0101, 0301, 0401, 0701, 0801, 1101, 1301, 1501) that "cover" the genetic backgrounds of most humans worldwide [28]. The analysis is performed by breaking peptide *p *into a set of overlapping 9mers, and assessing each such 9mer *e *for both MHC binding to each allele *a *ϵA, *F*_MHC_(*e*;*a*), and also conditional TCR recognition of the epitope: MHC complex, *F*_TCR|MHC_(*e*;*a*) if *e *is an MHC binder. Then *J*(*p*; *A*) combines information across alleles and epitopes.

#### MHC binding: *F*_MHC_(*e*;*a*)

EpiMatrix is a pattern-matching algorithm used for identification and prediction of T cell epitopes [29]. EpiMatrix evaluates binding potential of every 9mer in a protein sequence to the eight common class II HLA alleles listed above. EpiMatrix raw scores are normalized to Z-scores. Peptides with Z scores above 1.64 comprise the top 5%, and it is these peptides that are defined as "hits" and considered potentially immunogenic.

**Figure 2:**
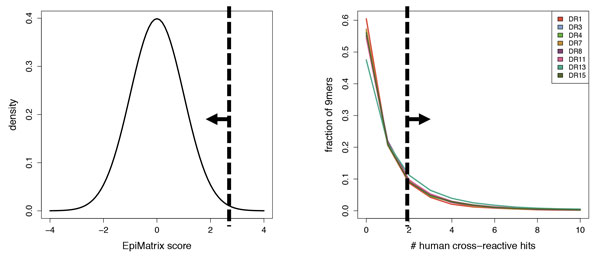
**Components of the Janus Immunogenicity Score**. JIS combines a prediction of MHC binding with a prediction of TCR binding conditioned on MHC binding. (left) MHC binding is predicted by EpiMatrix, which makes an allele-specific assessment of binding relative to a Normal distribution. The JIS contribution is the cumulative probability for those peptides exceeding a given threshold. (right) MHC-conditioned TCR binding is analyzed by JanusMatrix, which makes an allele-specific assessment of how many peptides in the human genome are predicted to bind the same MHC (possibly with different amino acids in the MHC pockets) and present the same amino acids to the TCR. The JIS contribution is the tail probability with respect to this empirical distribution. The plot is truncated at 10.

For an epitope *e *that is predicted to bind allele *a *(i.e., meeting the threshold), *F*_MHC_(*e*;*a*) is defined as the cumulative value in the Normal distribution for the Z-score (Figure 2, left) - a higher Z-score indicates a higher probability to be immunogenic. For a predicted non-binder against allele *a*, *F*_MHC_(*e*;*a*) is undefined. Except for one illustrative discussion point, the 5% threshold is used throughout the results.

#### TCR recognition conditioned on MHC binding: *F*_TCR|MHC_(*e*;*a*)

JanusMatrix [27] examines T cell epitopes, predicted by EpiMatrix, identifying as potentially cross-reactive those that are predicted to bind the same MHC (though perhaps with different amino acid composition on the MHC-facing side) while presenting the same amino acids to the TCR. JanusMatrix searches for such potentially cross-reactive TCR-facing epitopes across different sequence databases.

Here the extent of epitope cross-reactivity is assessed against the human genome (UniProt reviewed [30]). Note that the evaluation is conditional; only predicted MHC binders are considered. An empirical distribution is employed to calibrate the expected number of cross-reactive hits and convert the observed number into a probabilistic score. In particular, a 1,000,000 residue pseudoprotein, randomly generated at a natural amino acid frequency composition [31], was broken into overlapping 9mers and assessed for predicted epitopes binding to one or more alleles and for human genome cross-reactivity of those epitopes. *F*_TCR|MHC_(*e*;*a*) is then the tail probability in this empirical distribution (Figure 2, right) - fewer cross-reactive hits implies a higher probability of being immunogenic.

#### Integrated Janus Immunogenicity Score: *J*(*p*; *A*)

Let *E *be the set of 9mer epitopes within *p *that are predicted to bind one or more alleles. Each such epitope *e *is evaluated against each allele *a *in *A*. A joint probability of MHC binding and conditional TCR binding ensures that both sides of the epitope sandwich are satisfied - the combination of good MHC and good TCR binding implies a higher probability of being immunogenic. A joint probability over the alleles *A *encodes the notion that a promiscuous epitope is more likely to be immunogenic [32]. Alleles are assumed to be independent, from a distinct, representative set, so the joint probability is simply the product of the probabilities; to avoid numerical problems with small numbers, sums of log probabilities are employed in practice. Since the scores are only defined for epitopes, a uniform penalty term is incorporated as the contribution (in place of the log joint probability) for the alleles for which an epitope is a non-binder. The results presented use log 0.5 as this penalty, though the same trends were observed with other choices. The average is taken over all the epitopes (predicted binders) in the peptide, treating each independently (assuming they are presented separately) and uniformly (though an assessment of processing probability could readily be incorporated). In summary, the Janus immunogenicity score *J *is computed as:

$$J\left(p;A\right)=\frac{1}{\left|E\right|}\underset{e\in E}{\sum }\underset{a\in A}{\sum }\log\left(F_{\text{MHC}}\left(e;a\right)\cdot F_{\text{TCR}|\text{MHC}}\left(e;a\right)\right)$$

### Teff phenotype data

T cell data (ELISA, ELISPOT, and ICS) for five relevant cytokines (IFNγ, IL-4, IL-17, TGFβ, and IL-10) MHC-typed to one of the eight representative alleles listed above was downloaded from the IEDB [33]. A peptide was classified Teff-pos if any of the cytokines IFNγ, IL-4, or IL-17 was reported with a Positive measurement and neither of the cytokines TGFβ and IL-10 was; else it was classified Teff-neg/null.

### Viruses

To study the two extremes of viral camouflage, four viruses from the UniProt reviewed set were selected: Epstein-Barr virus (EBV; strain AG876) and cytomegalovirus (CMV; strain Merlin) were selected to serves as representatives of long-lived infection viruses, and Ebola (strain Mayinga-76) and Marburg (strain Musoke-80) were selected to serve as representatives of short-lived/zoonotic viruses. The JIS for each overlapping 15mer "fragment" was computed for each virus; 15mer fragments with no predicted epitopes were separately noted. As a control for the zoonotic extreme, a set of 73 plant-host viruses from [34] was likewise processed, with the entire set of fragments collected in aggregate.

For a more diverse set of human-host viruses, the all-fragment analysis was performed on all 51 human-host viruses [34]. Each virus was characterized by the median JIS in the distribution over all its fragments, treating non-epitopes as the lowest value in the distribution.

### Flu proteins

To study differences among related strains of the same virus, JIS was computed for each 15mer fragment in three representative influenza A strains: H1N1 (A/California/07/2009), the most recent pandemic strain; H5N1 (A/Goose/Guangdong/1/1996 H5N1 genotype Gs/Gd), a representative H5 avian influenza strain that has been responsible for many human deaths; and H7N9 (A/Shanghai/2/2013), also a recently emerged avian strain that caused more than 100 infections and 44 deaths in 2013. The JIS distribution was evaluated separately for the fragments in each of the two surface proteins hemagglutinin (HA) and neuraminidase (NA), as well other common proteins: nucleoprotein (NP), matrix proteins (M1 and M2), and non-structural proteins (NS1 and NEP).

## Results and discussion

### Teff phenotype characterization

A total of 588 peptides with Teff-pos peptide responses and 76 with Teff-neg/null responses were identified from the IEDB. To assess whether JIS is characteristically different for Teff-pos and Teff-neg/null, the distributions of scores for each set were separately characterized. The epitope predictor threshold separating MHC binders vs. non-binders is typically set to control the false positive rate (by taking only the most confident predictions), but it also naturally impacts the false negative rate. In this dataset, the standard 5% threshold eliminated 11% of the Teff-pos peptides and 20% of the Teff-neg/null peptides as having no predicted epitopes, while a more relaxed 10% threshold eliminated 4% and 5%, respectively. While these data points could be considered false negatives for immunoinformatics analysis, they might also be false positives in the T cell assay. To be consistent with standard practice and keep false positive rate low, the 5% threshold was employed for the remainder of the analysis.

**Figure 3:**
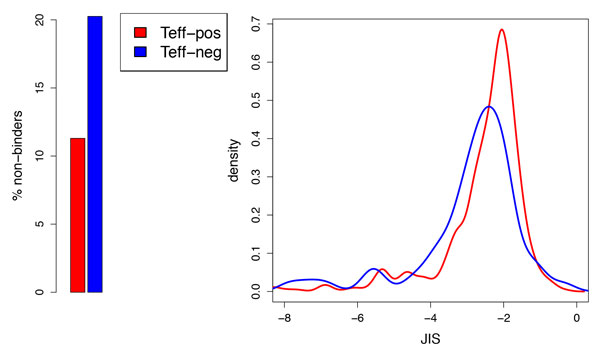
**JIS of IEDB peptides determined to be Teff-pos vs. those determined to be Teff-neg/null**. (left) Percentage of peptides that are predicted non-binders for all alleles; (right) JIS distributions (smoothed by kernel density estimation) of peptides predicted to bind at least one allele, truncated at a minimum of -8.

Figure 3 illustrates the fraction of non-binders and the JIS distributions for the Teff-pos and Teff-neg/null peptides. The Wilcoxon rank-sum test indicates that the Teff-neg/null distribution is "less than" the Teff-pos one (i.e. predicted to be less immunogenic overall), with a p-value of .0002 when including non-binders in the test, giving them an arbitrary lowest score, and .002 when testing just the binders. Thus we conclude that JIS does indeed distinguish immunogenicity risk, separating T cell epitopes that induce regulatory cytokines or no detected immune response from those that are associated with T effector cytokines, for this dataset.

**Figure 4:**
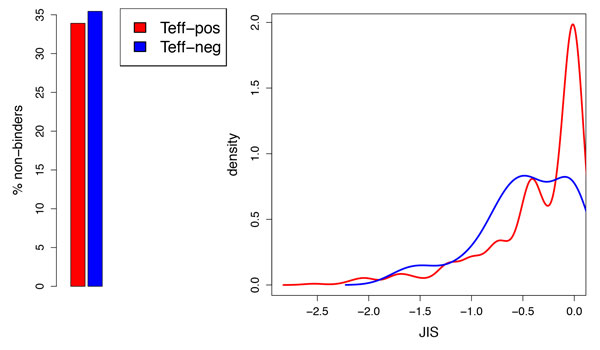
**JIS of IEDB peptides determined to be Teff-pos vs. those determined to be Teff-neg/null with JIS using only the allele tested in the experiment**. (left) Percentage of peptides that are predicted non-binders for the tested allele; (right) JIS distributions (kernel density estimation) of peptides predicted to bind the tested allele.

While JIS is computed over the eight standard alleles (capturing increased likelihood of immunogenicity for promiscuous binders), most peptides in the IEDB were tested for only one allele. The dataset includes 646 allele-specific characterizations for the 588 Teff-pos peptides and 79 for the 76 Teff-neg/null ones. Computing JIS for just the tested allele (ignoring the fact that other alleles, if tested, might be consistent) results in the distributions illustrated in Figure 4. There is no longer a distinction between Teff-pos vs. Teff-neg/null non-binders, and thus the Wilcoxon p-value for the entire set increases to .06. However, the distributions of the binders are still significantly different, at a p-value of .008, and there is a striking peak in the distribution at around 0 (i.e., a log-probability of 1) for Teff-pos peptides. Thus even in this weaker test (throwing away valuable predictive features due to sparse testing), we conclude that JIS is highly informative regarding immunogenicity, revealing a clear difference between pathogen-derived peptides that are reported as being associated with effector cytokines and those that are reported to be associated with Treg or null (absent) immune responses.

### Chronic vs. acute viruses

**Figure 5:**
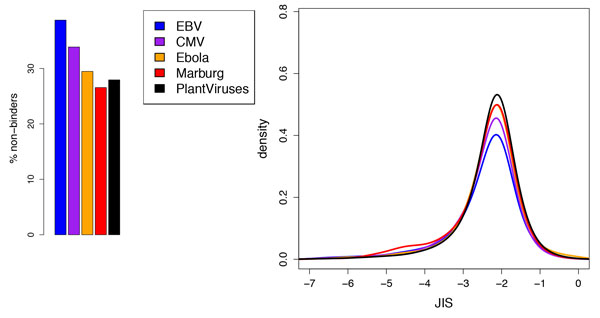
**JIS of 15mer fragments for representative viruses of different infection types**. (left) Percentage of fragments predicted to be non-binders; (right) JIS distributions (kernel density estimation, truncated at -7) of predicted binders; differences in total areas are due to different percentages of non-binders.

As Figure 5 illustrates, there are strong differences in the JIS distributions for the 15mer fragments in hit-and-stay viruses vs. those in the hit-and-run ones. EBV and CMV are predicted to have a relatively larger percentage of non-binder fragments and less immunogenicity for the binder fragments, while Ebola and Marbug (lines substantially overlapping in the mode of the distribution) are predicted to have more immunogenic fragments. Plant-host viruses (which do not infect humans) were used as a control, since these viruses can be assumed not to have any significant evolutionary contact with the human immune system. This set of viruses has a lower percentage of binders than Marburg, but higher JIS. Wilcoxon rank-sum tests, again treating non-binders as the lowest score, support the observed order EBV < CMV < Ebola = Marburg < plant-host viruses, with p-values approximately 0 for EBV vs. others and CMV vs. others, 2*10^-6 ^for Ebola vs. plant-host viruses, and 6*10^-4 ^for Marburg vs. plant-host viruses. Most of these relationships hold even among just the binders, indicating a significant shift in the quantitative scores, though the EBV vs. CMV p-value increases to .03 and EBV vs. Marburg to .01; CMV vs. Marburg crosses the usual line up to .06. Thus we conclude that the immunogenicity profiles of commensal hit-and-stay viruses are significantly different from hit-and-run viruses that cause acute disease and high mortality, in two ways. Commensal viruses appear to avoid immune response by (1) reducing their epitope content and (2) adopting more human-like TCR faces among the remaining epitopes, when compared to acute viruses and plant-virus controls.

### Broad set of human-host viruses

**Figure 6:**
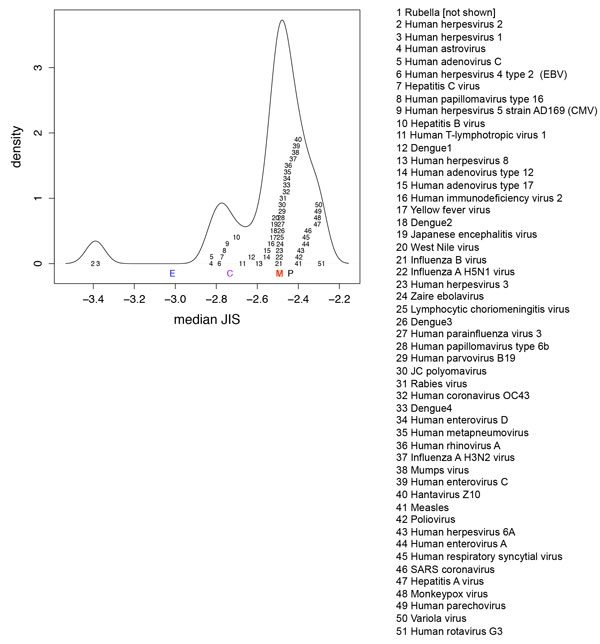
**Median JIS over all 15mer fragments, treating predicted non-binders as the lowest JIS, for each of a set of different human-host viruses**. The medians of the representative viruses EBV, CMV, Marburg, Ebola are indicated at the bottom, along with the median over all fragments in the plant-host virus set. The additional viruses are plotted at their median JIS, staggered in *y *value. Rubella is not shown, as over 50% of its peptides are non-binders. The curve provides a kernel density estimation of the median JIS distribution.

**Figure 7:**
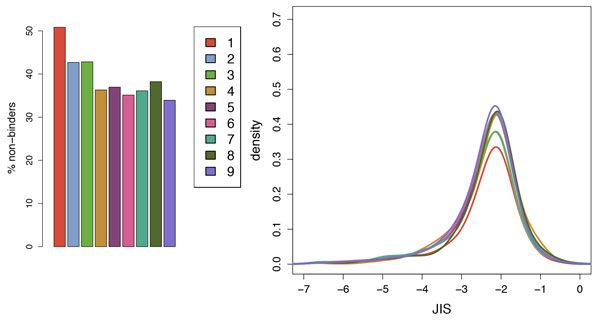
**JIS for viruses with low median JIS**. Numbers in the legend correspond to the indices in Figure 6. (left) Percentage of fragments predicted to be non-binders; (right) JIS distributions (kernel density estimation, truncated at -7) of predicted binders.

Figure 6 plots the median JIS among the fragments for each virus, with the chronic vs. acute case study viruses plotted at the bottom and the broad set stacked up above them (at arbitrary *y *coordinates to avoid overlap). Most of those on the low JIS side (various herpesviruses including a different EBV; astrovirus; heptatitis C virus) do indeed chronically infect humans and thus face evolutionary pressure to camouflage themselves reducing immune response. Figure 7 details the non-binder fractions and JIS distributions of the viruses with median JIS in the same range as the representative chronic viruses EBV and CMV. We see that this broader set of viruses also appear to have evolved sequences that contained reduced T cell epitope content and increased human cross-reactivity at the TCR facing residues of T cell epitopes (stronger low-JIS tail in the distribution).

**Figure 8:**
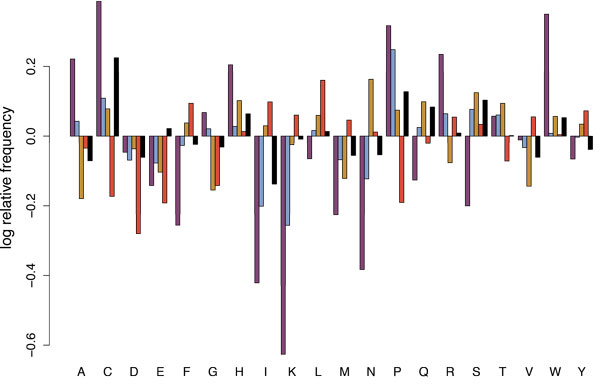
**Relative amino acid content for selected viral genomes, a large set of random predicted epitopes, and the human genome**. The fraction of each amino acid type in each genome (or set of epitopes) is divided by the corresponding fraction in a background set of proteins. The plot illustrates the log_10 _relative frequencies, so that overrepresented amino acids are and positive and underrepresented ones are negative.

The striking outlier in this analysis is rubella, for which a substantial fraction (50.8%) of fragments contain no predicted binders and thus which cannot be shown in the median-JIS plot. In order to better understand why this virus exhibits this pattern, and how that relates to some of the other representative viruses, the overall amino acid content within the different genomes was characterized. Figure 8 plots the log of the fraction of some such genomes, relative to that of a background set of proteins [31]. It also shows the corresponding values for the pseudoepitopes used to establish the empirical distribution for *F*_TCR|MHC_(*e*;*a*) as well as values for human genome. As is well-known (and encapsulated in epitope predictors), T cell epitopes are enriched in hydrophobic residues (F, I, L, M, V); the plot shows that in this dataset, the enrichment comes particularly at the expense of negatively charged residues (D, E) as well as a few others (C, G, P). The acute Marburg has similar trends, while the chronic EBV is less consistent and even reverses some of them. Human also reverses some of the trends. Rubella goes against the trends to an extreme degree, often in the same direction as human and away from binders, but much further. Quantitatively, the Euclidean distance from the background frequency distribution to the Rubella one is .117, while the distance from background to EBV is .057, to Marburg .058, to binders .067, and to human .037. Rubella is even further away from the set of binders, at a distance of .153; thus, Rubella has strikingly different amino acid prevalence. While the implication of this finding is uncertain, it may relate to an as-yet undescribed means of immune escape. In future studies we plan to identify pathogens that have similar amino acid distributions and examine their relationship with human hosts.

### Influenza proteins

**Figure 9:**
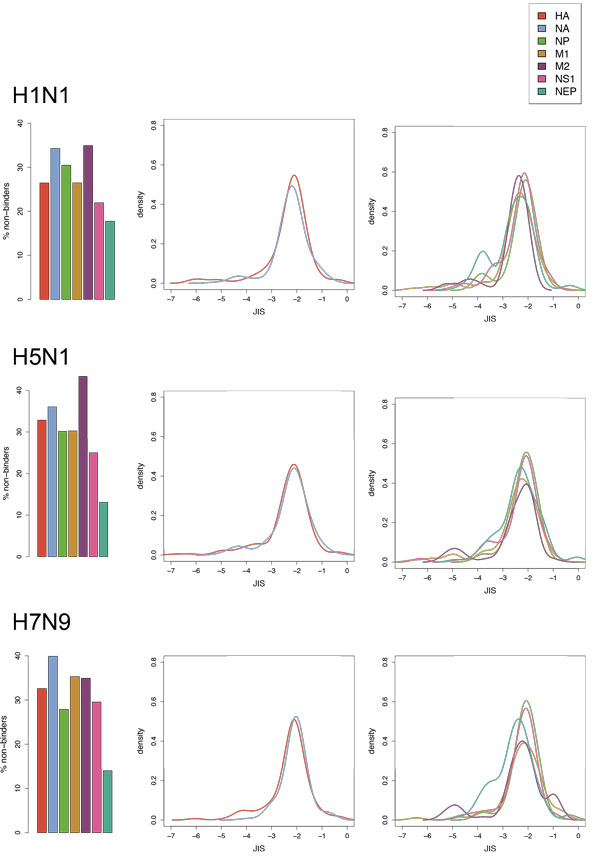
**JIS distributions over 15mer fragments in individual proteins in three influenza A strains**. (left) Percentage of fragments predicted to be non-binders; (middle, right) JIS distributions (kernel density estimation, truncated at -7) of predicted binders for common proteins, with surface proteins separated from others.

For comparisons within virus species, JIS distributions can be separately characterized for different proteins. Using influenza as a case study, Figure 9 illustrates such per-protein distributions for three different strains, separating out the surface antigens HA and NA from the others. As would be expected, in general HA and NA are relatively de-immunized and somewhat shifted toward lower JIS (which would reduce their overall ability to induce antibody responses). In contrast, NP and NS1 have the biggest concentration of high-JIS epitopes, while NEP's broader JIS distribution suggests that it has more human-like fragments than those. M2 is strikingly de-immunized, a fact we attribute to a notably different amino acid content supporting its structure and function as a proton channel, with specific patterns for formation of a transmembrane homotetramer establishing the pore, and a large number of E in the C terminus for complex formation with M1 [35]).

In contrasting the different strains, H7N9 is generally more deimmunized than H5N1 and H1N1, and is for both HA and NA in particular. HA5 is also more deimmunized than HA1. The low immunogenicity scores of H7N9 has been previously reported by our group. The significance of this finding is unknown, since neither virus would have normally interacted with the human immune system; this finding may be relevant to immune responses that are directed against the virus by its other hosts (birds and swine).

## Conclusions

While cross-reactivity is now understood as an intrinsic characteristic of the T cell receptor, critical to training and protection, this paper presents the first attempt to quantitatively evaluate it, and to use such a characterization in a genomic-scale study of how pathogens may use this means of escaping an immune response. Our model unifies into the novel Janus Immunogenicity Score both sides of recognition: MHC-based epitope prediction and extent of TCR-level cross-reactivity against self peptides (conditioned on MHC binding). The predictive power of the JIS is demonstrated by comparing epitopes in the IEDB that are associated with T effector cytokines ("Teff-pos") vs. those that are associated with regulatory T cell cytokine profiles or lack of immune response ("Teff-neg/null"), and finding a statistically significant difference in scores.

With this immunogenicity score in hand, we studied profiles of viruses associated with chronic infection (hit-and-stay) vs. those that cause acute disease and higher mortality (hit-and-run), and found them to be significantly different. Commensal viruses appear to avoid immune response by (1) reducing their epitope content and (2) adopting more human-like TCR faces among the remaining epitopes, when compared to acute viruses and plant-virus controls. When expanding the analysis to a broader set of viruses, several of these also appear to have evolved sequences that contained reduced T cell epitope content and increased human cross-reactivity at the TCR facing residues of T cell epitopes (stronger low-JIS tail in the distribution). Rubella is a striking outlier, which may relate to an as-yet undescribed means of immune escape. In future studies we plan to identify pathogens that have similar amino acid distributions and examine their relationship with human hosts.

And finally, among influenza viruses, H7N9 is generally more deimmunized than H5N1 and H1N1, and is for both HA and NA in particular. HA5 is also more deimmunized than HA1. The low immunogenicity scores of H7N9 has been previously reported by our group. The significance of this finding is unknown, since neither virus would have normally interacted with the human immune system; this finding may be relevant to immune responses that are directed against the virus by other hosts (birds and swine).

## List of abbreviations used

APC: antigen presenting cell; CMV: cytomegalovirus; EBV: Epstein-Barr virus; IEDB: immune epitope database; JIS: Janus immunogenicity score; MHC: major histocompatibility complex; TCR: T cell receptor

## Competing interests

Coauthors ADG, WM and LM are employees of EpiVax, a vaccine and therapeutic design company, and two (ADG, WM) are majority stockholders. These authors recognize the presence of potential conflicts of interest and affirm that the information represented in this paper is original and unbiased observations. In addition to his role as a faculty member at Dartmouth, CBK is co-founder and CTO of Stealth Biologics, LLC, a therapeutic protein design company. Dartmouth has worked with him to manage all potential conflicts of interest arising from his commercial affiliation, and he likewise affirms that this paper presents work free of any bias. LH and ANG declare no competing interests.

## Authors' contributions

LH, ADG, WM, and CBK conceived of the idea; LH and CBK designed the methods with feedback from the others; LH implemented the methods and collected the results; LH and CBK analyzed the results with feedback from the others; LH, ADG, and CBK wrote the paper. All authors read and approved the final manuscript.

## Declarations

The publication of this work was funded by NSF grant IIS-1017231 to CBK.

This article has been published as part of BMC Bioinformatics Volume 15 Supplement 4, 2014: Selected articles on Computational Vaccinology 2013. The full contents of the supplement are available online at http://www.biomedcentral.com/bmcbioinformatics/supplements/15/S4.
